# Implication of the σ^E^ Regulon Members OmpO and σ^N^ in the Δ*ompA_299–356_*-Mediated Decrease of Oxidative Stress Tolerance in Stenotrophomonas maltophilia

**DOI:** 10.1128/spectrum.01080-23

**Published:** 2023-06-07

**Authors:** Ren-Hsuan Ku, Li-Hua Li, Yi-Fu Liu, En-Wei Hu, Yi-Tsung Lin, Hsu-Feng Lu, Tsuey-Ching Yang

**Affiliations:** a Department of Biotechnology and Laboratory Science in Medicine, National Yang Ming Chiao Tung University, Taipei, Taiwan; b Department of Pathology and Laboratory Medicine, Taipei Veterans General Hospital, Taipei, Taiwan; c School of Medical Laboratory Science and Biotechnology, College of Medical Science and Technology, Taipei Medical University, Taipei, Taiwan; d Division of Infectious Diseases, Department of Medicine, Taipei Veterans General Hospital, Taipei, Taiwan; e Department of Medicine, National Yang Ming Chiao Tung University, Taipei, Taiwan; f Department of Medical Laboratory Science and Biotechnology, Asia University, Taichung, Taiwan; University of Manitoba

**Keywords:** OmpA, RpoN, sigma factor, outer membrane proteins

## Abstract

Outer membrane protein A (OmpA) is the most abundant porin in bacterial outer membranes. KJΔOmpA_299–356_, an *ompA* C-terminal in-frame deletion mutant of Stenotrophomonas maltophilia KJ, exhibits pleiotropic defects, including decreased tolerance to menadione (MD)-mediated oxidative stress. Here, we elucidated the underlying mechanism of the decreased MD tolerance mediated by Δ*ompA_299–356_*. The transcriptomes of wild-type S. maltophilia and the KJΔOmpA_299–356_ mutant strain were compared, focusing on 27 genes known to be associated with oxidative stress alleviation; however, no significant differences were identified. *OmpO* was the most downregulated gene in KJΔOmpA_299–356_. KJΔOmpA_299–356_ complementation with the chromosomally integrated *ompO* gene restored MD tolerance to the wild-type level, indicating the role of OmpO in MD tolerance. To further clarify the possible regulatory circuit involved in *ompA* defects and *ompO* downregulation, σ factor expression levels were examined based on the transcriptome results. The expression levels of three σ factors were significantly different (downregulated levels of *rpoN* and upregulated levels of *rpoP* and *rpoE*) in KJΔOmpA_299–356_. Next, the involvement of the three σ factors in the Δ*ompA_299–356_*-mediated decrease in MD tolerance was evaluated using mutant strains and complementation assays. *rpoN* downregulation and *rpoE* upregulation contributed to the Δ*ompA_299–356_*-mediated decrease in MD tolerance. OmpA C-terminal domain loss induced an envelope stress response. Activated σ^E^ decreased *rpoN* and *ompO* expression levels, in turn decreasing swimming motility and oxidative stress tolerance. Finally, we revealed both the Δ*ompA_299–356_*-*rpoE*-*ompO* regulatory circuit and *rpoE*-*rpoN* cross regulation.

**IMPORTANCE** The cell envelope is a morphological hallmark of Gram-negative bacteria. It consists of an inner membrane, a peptidoglycan layer, and an outer membrane. OmpA, an outer membrane protein, is characterized by an N-terminal β-barrel domain that is embedded in the outer membrane and a C-terminal globular domain that is suspended in the periplasmic space and connected to the peptidoglycan layer. OmpA is crucial for the maintenance of envelope integrity. Stress resulting from the destruction of envelope integrity is sensed by extracytoplasmic function (ECF) σ factors, which induce responses to various stressors. In this study, we revealed that loss of the OmpA-peptidoglycan (PG) interaction causes peptidoglycan and envelope stress while simultaneously upregulating σ^P^ and σ^E^ expression levels. The outcomes of σ^P^ and σ^E^ activation are different and are linked to β-lactam and oxidative stress tolerance, respectively. These findings establish that outer membrane proteins (OMPs) play a critical role in envelope integrity and stress tolerance.

## INTRODUCTION

Reactive oxygen species (ROS) production, a natural consequence of aerobic metabolism, is inevitable in aerobic bacteria. Aerobic-metabolism-generated ROS mainly include hydrogen peroxide (H_2_O_2_), the superoxide radical (O_2_^–•^), and the hydroxyl radical (HO^•^). Pathogens also encounter oxidative stress challenges produced by the host immune system during infection (1). As a result, bacteria use various defense systems to manage oxidative stress (2). Oxidative stress arises due to an imbalance between ROS generation and elimination, causing significant damage to the bacterial cell envelope, lipids, and proteins. Oxidative stress defense systems are diverse and include enzymatic systems (superoxide dismutase [SOD], catalase [Kat], hydroperoxide reductase [Ahp], and glutathione peroxidase [Gpx]) (3), nonenzymatic antioxidants (NADPH, NADH, β-carotene, ascorbic acid, α-tocopherol, and glutathione), and efflux pumps (the EmrBA1-SilC pump of Francisella tularensis, the SCO4121 pump of Streptomyces coelicolor, and the MacAB pump of Salmonella enterica serovar Typhimurium) (4–6). Bacteria can coordinate complex regulatory systems, such as transcriptional regulators, two-component regulatory systems, small RNAs, and σ factors, to generate an effective oxidative stress response (7–15).

Gram-negative bacterial cells are encompassed by a cell envelope that protects them from external stimuli (16). Furthermore, the envelope serves as a gateway for the import and export of various compounds. The envelope stress response (ESR) represents a complex regulatory network that monitors envelope integrity and mounts an appropriate response to mitigate stress (17). The best-characterized ESR consists of the σ^E^-RseA-RseB pathway in Escherichia coli and most *Gammaproteobacteria* (18). The extracytoplasmic σ factor, σ^E^, is a master regulator of the ESR (19). In normal E. coli cells, σ^E^ is mostly inactive owing to RseA sequestration. RseA is an inner membrane anti-σ factor with a cytoplasmic domain that binds to σ^E^ to maintain it in an inactive state (20). Under envelope stress, RseA is degraded by two inner membrane proteases, DegS and RseP, and then by the cytoplasmic protease, ClpXP, subsequently releasing free σ^E^ (21, 22). RseB functions as a negative regulator of the σ^E^ pathway by binding RseA to increase the affinity of the RseA-RseB complex for σ^E^, in turn inhibiting the σ^E^ pathway (23). Known σ^E^ pathway inducers include misfolded outer membrane protein (OMP), periplasmic lipopolysaccharide accumulation, oxidative stress, heat shock, carbon starvation, biofilm formation, acid stress, UV-A radiation, P22 phage attack, and hypo-osmotic shock (24, 25).

RNA polymerase (RNAP), a DNA-dependent enzyme complex responsible for bacterial transcription, is composed of six subunits, namely, α, α, β, β′, ω, and σ (26). The σ subunit recognizes cognate promoter sequences and ensures adequate gene expression levels. Generally, bacteria harbor several different σ factors that can recognize their own cognate promoter sequences and ensure that all functional genes in the genome are expressed as required (27). σ factors are classified into two structurally and evolutionarily distinct families: σ^70^ and σ^54^ families. σ^70^ family members are further divided into four groups. Group 1 comprises primary or housekeeping σ factors; groups 2 and 3 comprise σ factors that regulate the expression of genes involved in general stress, flagellar structure and function, and chemotaxis; and group 4 (extracytoplasmic function [ECF]) comprises the largest group of σ factors that are involved in a major signaling network that enables bacteria to adapt to various environmental stimuli (28, 29). Bacteria typically harbor several ECF σ factors that induce adaptive responses to environmental changes. ECF σ factor expression and activity can be modulated at many levels but are predominantly controlled by association with the anti-σ factor. Upon signal perception, ECF σ factors are released from the anti-σ factor and assemble with the core RNA polymerase to induce gene expression. Genes encoding the ECF σ factors and their cognate anti-σ factor are located in the same operon (30).

Stenotrophomonas maltophilia, a nonfermenting Gram-negative rod bacterium, is prevalent in nature, in places such as soil, plant roots, animals, and aqueous environments. S. maltophilia is an opportunistic pathogen and a multidrug-resistant organism of concern in hospitals (31). Given its diverse habitats, S. maltophilia uses several stress alleviation systems to ensure survival in different environments. We previously reported several oxidative stress alleviation systems in S. maltophilia, including enzymatic (SOD, Kat, Ahp, Gpx), efflux pump (MasABCsm, SmeYZ, and SmeVWX), and formaldehyde detoxification (FadACB) systems (32–37). Furthermore, similar to the well-studied σ^E^-RseA system of E. coli (18), S. maltophilia harbors an *rpoE-rseA-mucD* operon (Smlt3555-3553) that is responsible for ESR (38).

Recently, we reported that *ompA* is highly expressed in logarithmic-phase S. maltophilia KJ cells (38). We characterized an in-frame deletion *ompA* mutant of S. maltophilia KJ, originally termed KJΔOmpA (39) and later renamed KJΔOmpA_299–356_ (40). We also reported that the truncated OmpA protein can be stably embedded in the outer membrane but loses contact with peptidoglycan (PG) (40). KJΔOmpA_299–356_ exhibits decreased conjugation ability and swimming motility (39), as well as increased susceptibility to β-lactams (40). Transcriptome analysis revealed that the expression levels of the three σ factors, *rpoN*, *rpoP*, and *rpoE*, are significantly altered in KJΔOmpA_299–356_ (39, 40). Notably, *rpoN* downregulation is the key factor contributing to the swimming defect in KJΔOmpA_299–356_, and upregulated σ^P^ is involved in the Δ*ompA_299–356_*-mediated increase in β-lactam susceptibility via the σ^P^-NagA-L1/L2 regulatory circuit (39, 40). Here, we aimed to further investigate the effect of Δ*ompA*_299–356_ on oxidative stress tolerance and elucidate the underlying mechanism.

## RESULTS

### KJΔOmpA_299-356_ is more susceptible than KJ to menadione.

**FIG 1:**
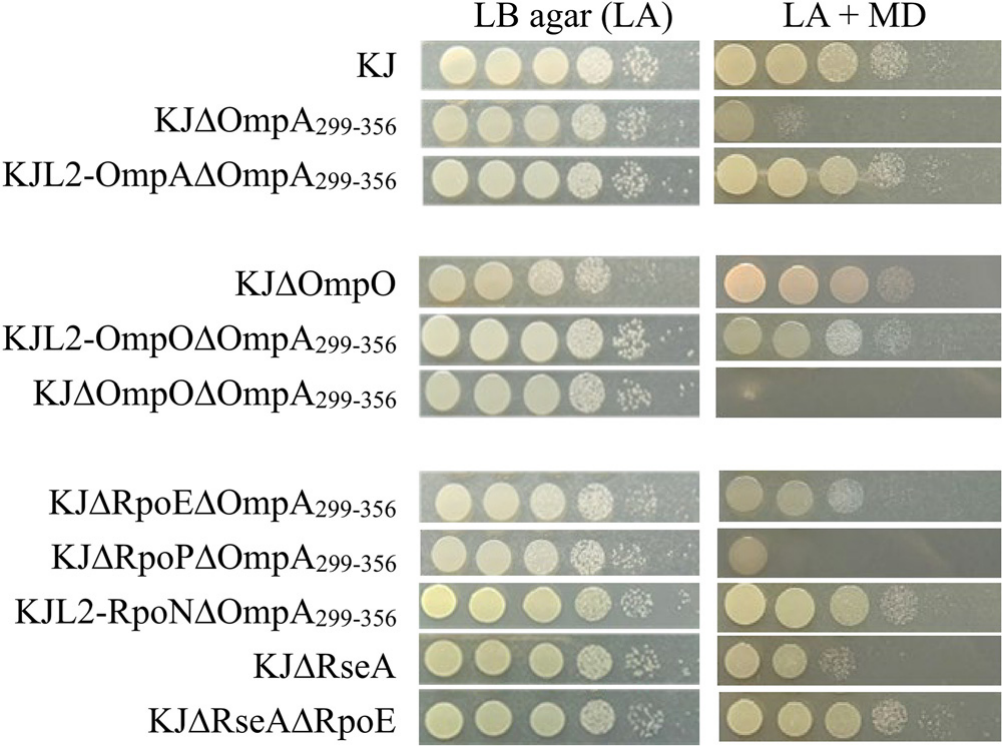
Menadione tolerance of wild-type KJ and its derived mutants. Logarithmic-phase bacterial cells (2 × 10^5^ CFU/μL) were 10-fold serially diluted. Then, 5-μL aliquots of the cells were spotted onto Luria-Bertani (LB) agar plates with and without 40 μg/mL menadione (MD). Bacterial growth was observed after 24 h of incubation at 37°C. All experiments were performed at least thrice, and one was selected as a representative experiment.

As KJΔOmpA_299–356_ loses its conjugation ability (39), a plasmid-mediated complementation assay using conjugation is not feasible. Therefore, we previously constructed an alternative *ompA* complementary strain, KJL2-OmpAΔOmpA_299–356_, in which the complemented *ompA* gene was inserted downstream of the *L2* gene and was driven by the *L2* promoter in KJΔOmpA_299–356_ (40). The impact of Δ*ompA*_299–356_ on oxidative stress tolerance was assessed by determining the cell viabilities of KJ, KJΔOmpA_299–356_, and KJL2-OmpAΔOmpA_299–356_ in menadione (MD)-containing media. Compared with wild-type KJ, the MD tolerance of KJΔOmpA_299–356_ was significantly compromised. However, MD tolerance nearly reverted to the wild-type level in the complementation strain, KJL2-OmpAΔOmpA_299–356_ (Fig. 1).

We previously characterized several oxidative stress alleviation systems in S. maltophilia, including enzymatic and nonenzymatic systems (32–37). To understand the mechanism involved in the Δ*ompA*_299–356_-mediated decrease in MD tolerance, we analyzed the transcriptome results of KJ and KJΔOmpA_299–356_ (39), focusing on genes involved in oxidative stress alleviation (see Table S1 in the supplemental material). A change in KJ and KJΔOmpA_299–356_ gene expression greater than 3-fold was considered statistically significant. Of the 27 genes analyzed, none exhibited significant alterations in transcript levels (Table S2), suggesting that an unidentified determinant is responsible for the Δ*ompA*_299–356_-mediated decrease in MD tolerance.

### *OmpO* (Smlt0387) expression is downregulated in KJΔOmpA_299–356_.

To further identify the putative candidates responsible for the Δ*ompA*_299–356_-mediated decrease in MD tolerance, we rechecked the transcriptome results (39), focusing on the top five upregulated and downregulated genes. *Smlt0387* was highly expressed in wild-type KJ and was the most downregulated gene (approximately a 203-fold decrease in expression levels) in KJΔOmpA_299–356_ (Table 1). Smlt0387 is annotated as a hypothetical protein in several sequenced S. maltophilia genomes. Based on the findings of this study, we designated Smlt0387 as OmpO. *OmpO* encodes a 190-amino acid (aa) protein. Subcellular location prediction (https://www.psort.org/psortb/) and signal peptide prediction (https://services.healthtech.dtu.dk/service.php?SignalP) indicated that OmpO was an OMP with a 19-aa signal peptide.

**TABLE 1:** Selected transcriptome analysis of wild-type KJ and *ompA* mutant, KJΔOmpA_299–356_

Locus	Protein	TPM^*a*^		Fold change^*b*^
		KJ	KJΔOmpA_299–356_	
Upregulated genes				
Smlt0603	Carboxypeptidase A	2.86	47.24	+16.55
Smlt0602	TonB-dependent receptor	11.79	195.12	+16.54
Smlt3952	Reductase	0.98	11.00	+11.20
Smlt3740	TonB-dependent receptor	40.37	430.30	+10.66
Smlt0564	Hypothetical protein	35.37	313.24	+8.86
Smlt3555	σ^E^	434.09	2,424.29	+5.58
Smlt3514	σ^P^	49.65	156.39	+3.15
Downregulated genes				
Smlt0387	OmpO	**5690.31** ^*c*^	**27.95**	**−203.56**
Smlt2730	Secretory pathway protein	3.33	0.19	−17.91
Smlt1149	Hypothetical protein	31.53	2.06	−15.34
Smlt2317	Proximal rod protein	26.61	2.19	−12.16
Smlt2283	Flagellar basal body protein	12.18	1.17	−10.38
Smlt2297	σ^N^	65.24	12.27	−5.32

a TPM, transcripts per kilobase million.

b Negative fold changes represent genes that were significantly downregulated in KJΔOmpA_299–356_, whereas positive fold changes represent upregulation in KJΔOmpA_299–356_.

c The bolded values indicate the most downregulated gene in KJΔOmpA_299–356_.

**FIG 2:**
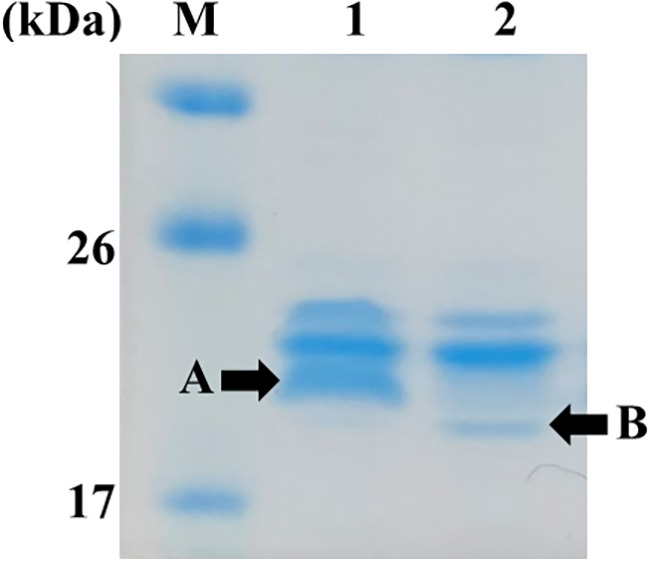
Outer membrane protein profiling of KJ and KJΔOmpO. Outer membrane proteins (OMPs) were prepared from logarithmic-phase KJ and KJΔOmpO and separated via sodium dodecyl sulfate-polyacrylamide gel electrophoresis (SDS-PAGE) with 5% stacking and 15% separating gels. Lane M, molecular weight standards; lane 1, KJ; lane 2, KJΔOmpO. A and B indicate the proteins that were excised for liquid chromatography-tandem mass spectrometry (LC-MS/MS) analysis.

To verify whether OmpO is indeed an OMP, the OMP profiles of wild-type KJ and KJΔOmpO, an *ompO* isogenic mutant, were analyzed via sodium dodecyl sulfate-polyacrylamide gel electrophoresis (SDS-PAGE). KJΔOmpO lacked a protein band that was present in KJ (band A in Fig. 2). This band was excised from the gel and characterized using liquid chromatography-tandem mass spectrometry (LC-MS/MS). The LC-MS/MS results correlated band A with Smlt0387 (OmpO). The expected mature OmpO protein had an expected molecular weight of 19.0 kDa, which matches the location of band A in the gel (Fig. 2). Furthermore, the fragmentation patterns of band A showed the absence of the predicted signal peptide (1 to 19 aa residues) (Table S3), confirming that OmpO is an OMP with a 19-aa signal peptide. Interestingly, another protein with a molecular weight smaller than OmpO was upregulated in KJΔOmpO (band B in Fig. 2). This band was characterized by LC-MS/MS, and the results correlated band B with Smlt0184 (Table S4).

**FIG 3:**
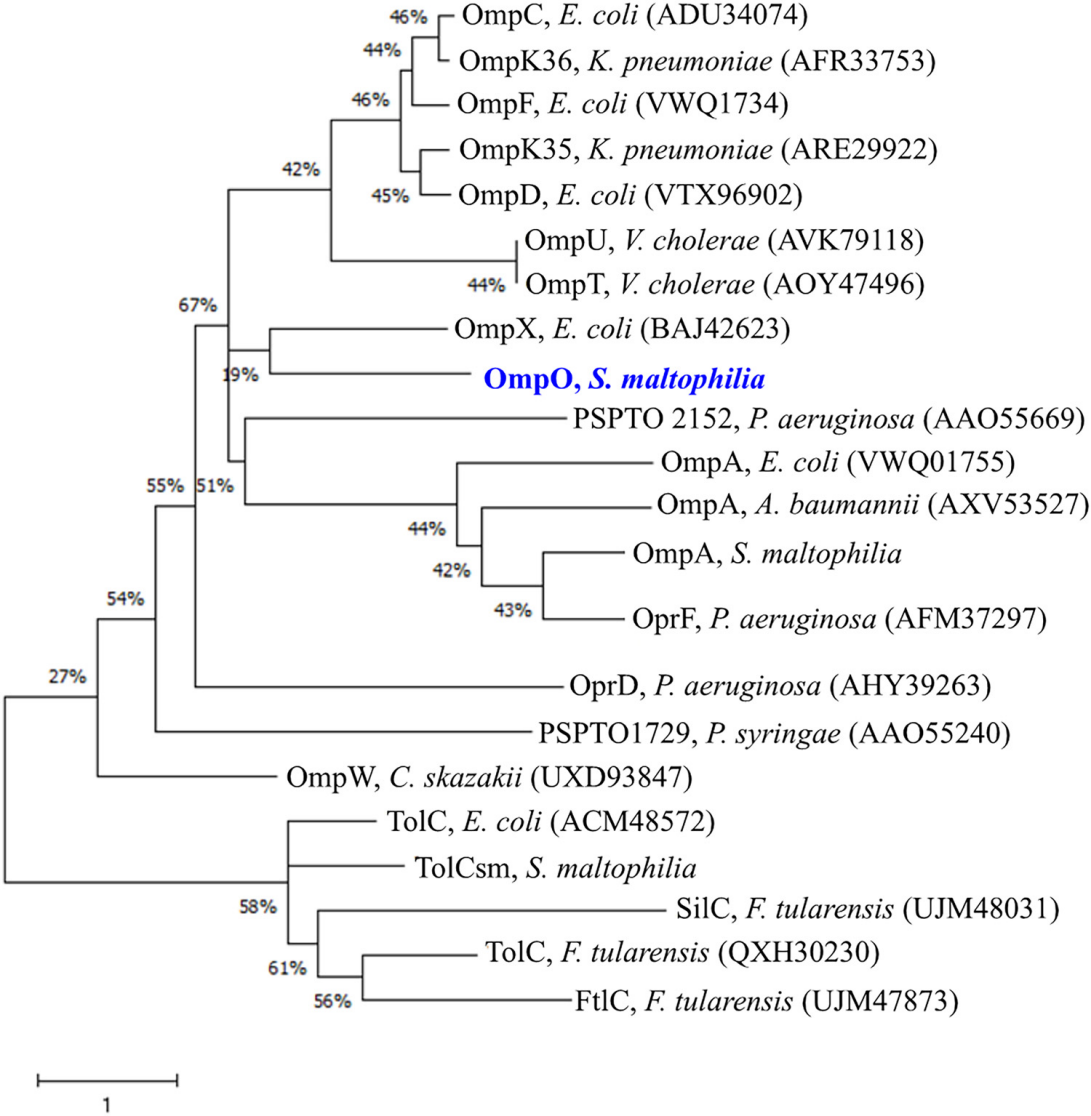
Phylogenetic analysis of OmpO of Stenotrophomonas maltophilia KJ and comparison with other OMPs. A phylogenic tree was constructed using the neighbor-joining method. Numbers at the branch nodes indicate the bootstrap values as a percentage of 1,000 replications. Accession numbers of the proteins are provided in parenthesis.

To further clarify the biological significance of OmpO, phylogenetic analysis of OmpO and other OMPs, including TolC family and β-barrel proteins (OmpA, OmpX, phospholipase A, general porins, substrate-specific porins, and TonB-dependent transporters), was conducted. OmpO was phylogenetically closely related to OmpX of E. coli (Fig. 3).

### Downregulated *OmpO* expression in KJΔOmpA_299–356_ is involved in the Δ*ompA_299–356_*-mediated decrease in MD tolerance.

To clarify the role of downregulated *ompO* expression in the Δ*ompA_299–356_*-mediated decrease in MD tolerance, the *ompO* allele was chromosomally integrated downstream of the *L2* gene of KJ cells to form KJL2-OmpO. The Δ*ompA_299–356_* allele was then introduced into the chromosome of KJL2-OmpO, generating KJL2-OmpOΔOmpA_299–356_. L2 and the integrated *ompO* gene formed an operon-mimic structure; therefore, *ompO* expression was driven by the *L2* gene promoter. *L2* expression is upregulated in KJΔOmpA_299–356_ (39); therefore, KJL2-OmpOΔOmpA_299–356_ can be an *ompO* complementation construct of KJΔOmpA_299–356_. We further constructed an *ompO* deletion mutant of KJΔOmpA_299–356_, KJΔOmpOΔOmpA_299–356_, and determined its MD tolerance capacity. Compared with the parental strain, KJΔOmpA_299–356_, KJL2-OmpOΔOmpA_299–356_ showed an MD tolerance level reverted to near the wild-type level, whereas KJΔOmpOΔOmpA_299–356_ exhibited low MD tolerance (Fig. 1), indicating that the extent of MD tolerance is proportional to the expression level of *ompO*. Therefore, OmpO downregulation in KJΔOmpA_299–356_ is involved in the Δ*ompA_299–356_*-mediated decrease in MD tolerance. As OmpO is related to oxidative stress tolerance, we designated Smlt0387 as OmpO in this study.

The involvement of OmpO levels in MD tolerance was revealed in the Δ*ompA_299–356_* genetic background, and we wondered whether a similar effect could be observed in the wild-type KJ genetic background. Thus, we prepared an *ompA* deletion construct of KJ cells for an MD tolerance assay. KJΔOmpO displayed MD tolerance comparable to that of its parent strain, KJ (Fig. 1).

### Upregulated *RpoE* (*Smlt3555*) and downregulated *rpoN* (*Smlt2297*) levels in KJΔOmpA_299–356_ are involved in the Δ*ompA*_299–356_-mediated decrease in MD tolerance.

We recently demonstrated that the expression levels of the three σ factors are significantly altered (upregulated σ^P^ and σ^E^ levels and downregulated σ^N^ levels) in KJΔOmpA_299–356_ (40). To assess whether the alteration of these σ factor expression levels is associated with the Δ*ompA*_299–356_-mediated decrease in MD tolerance, KJΔRpoEΔOmpA_299–356_, KJΔRpoPΔOmpA_299–356_, and KJL2-RpoNΔOmpA_299–356_, a strain of KJΔOmpA_299–356_ complemented with *rpoN* expression, were subjected to an MD tolerance assay. The MD tolerance assay revealed that KJΔRpoEΔOmpA_299–356_ and KJL2-RpoNΔOmpA_299–356_ had an MD tolerance level that was nearly reverted to the wild-type level, whereas KJΔRpoPΔOmpA_299–356_ exhibited MD tolerance comparable to that of KJΔOmpA_299–356_ (Fig. 1). These results indicated that *rpoE* upregulation and *rpoN* downregulation in KJΔOmpA_299–356_ are involved in the Δ*ompA*_299–356_-mediated decrease in MD tolerance.

The linkage between *rpoE* upregulation and the Δ*ompA*_299–356_-mediated decrease in MD tolerance highly suggested the occurrence of ESR in KJΔOmpA_299–356_. The involvement of the *rpoE-rseA* system in ESR has been characterized in several bacteria (18), including the *rpoE-rseA-mucD* operon in S. maltophilia (38). RseA functions as an anti-σ^E^ factor; thus, the loss of RseA function induces σ^E^ activation, mimicking ESR (18). To assess the relationship between ESR and MD tolerance, the MD tolerance assay was performed with KJΔRseA and KJΔRseAΔRpoE. Compared to wild-type KJ, KJΔRseA exhibited a decreased MD tolerance and KJΔRseAΔRpoE showed an MD tolerance level that had reverted to the wild-type level (Fig. 1), indicating that an ESR system reduces the resistance of bacteria to MD-induced oxidative stress.

### σ^E^ activation downregulates *ompO* expression.

**FIG 4:**
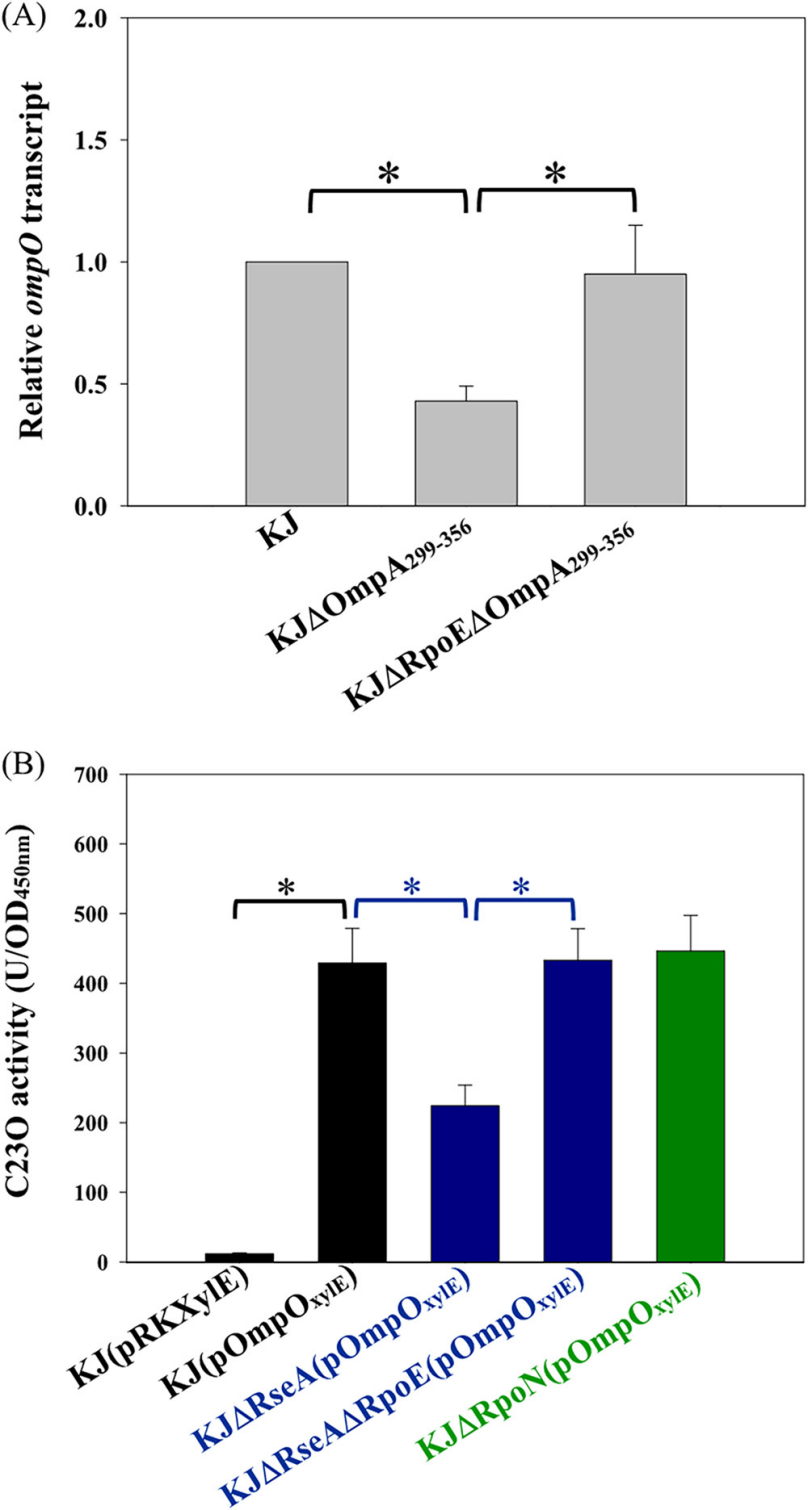
Roles of σ^E^ and σ^N^ in *ompO* expression. The data represent the means from three independent experiments. Error bars represent the standard deviations for triplicate samples. *, *P < *0.01, calculated via Student’s *t* test. (A) Impacts of σ^E^ activation on *ompO* expression in KJΔOmpA_299–356_. Overnight-cultured bacterial cells were inoculated into fresh LB with an initial optical density at 450 nm (OD_450_) of 0.15. After 5 h of aerobic culture, *ompO* transcript levels were determined by qRT-PCR. All values were normalized to the transcript levels of KJ cells. (B) Roles of σ^E^ and σ^N^ in *ompO* expression in wild-type KJ. Overnight cultures of tested S. maltophilia strains were inoculated into fresh LB medium at an initial OD_450_ of 0.15. After 7 h of culture, catechol 2,3-dioxygenase (C23O) activities were determined.

The next question was whether σ^E^ upregulation in KJΔOmpA_299–356_ imposes a negative effect on *ompO* expression. Thus, the *ompO* transcript levels in KJ, KJΔOmpA_299–356_, and KJΔRpoEΔOmpA_299–356_ were compared by quantitative reverse transcription PCR (qRT-PCR). The *ompO* transcript was downregulated in KJΔOmpA_299–356_ and reverted to near wild-type levels in KJΔRpoEΔOmpA_299–356_ (Fig. 4A). We further determined the impact of σ^E^ activation on *ompO* expression in the wild-type KJ. Plasmid pOmpO_xylE_, containing a *P_ompO_-xylE* transcriptional fusion construct, was introduced into KJ, KJΔRseA, and KJΔRseAΔRpoE to generate KJ(pOmpO_xylE_), KJΔRseA(pOmpO_xylE_), and KJΔRseAΔRpoE(pOmpO_xylE_), respectively. KJ(pOmpO_xylE_) exhibited significantly higher catechol 2,3-dioxygenase (C23O) activity than the vector-only control strain, KJ(pRKXylE) (Fig. 4), indicating that the *ompO* gene is highly expressed in logarithmic-phase KJ cells, consistent with the transcriptome results (Table 1). KJΔRseA(pOmpO_xylE_) showed lower C23O activity than KJ(pOmpO_xylE_). However, C23O activity was comparable in KJΔRseAΔRpoE(pOmpO_xylE_) and KJ(pOmpO_xylE_) (Fig. 4B), indicating that σ^E^ activation has a negative effect on *ompO* expression.

A similar strategy was used to investigate the regulatory effect of σ^N^ on *ompO* expression. The *rpoN* expression levels of KJ cells under our test condition were first verified by RT-PCR (data not shown). C23O activity was then determined in KJΔRpoN(pOmpO_xylE_). The results demonstrated that *rpoN* barely affected the expression level of the *ompO* gene (Fig. 4B).

### σ^E^ activation negatively regulates σ^N^ expression.

**FIG 5:**
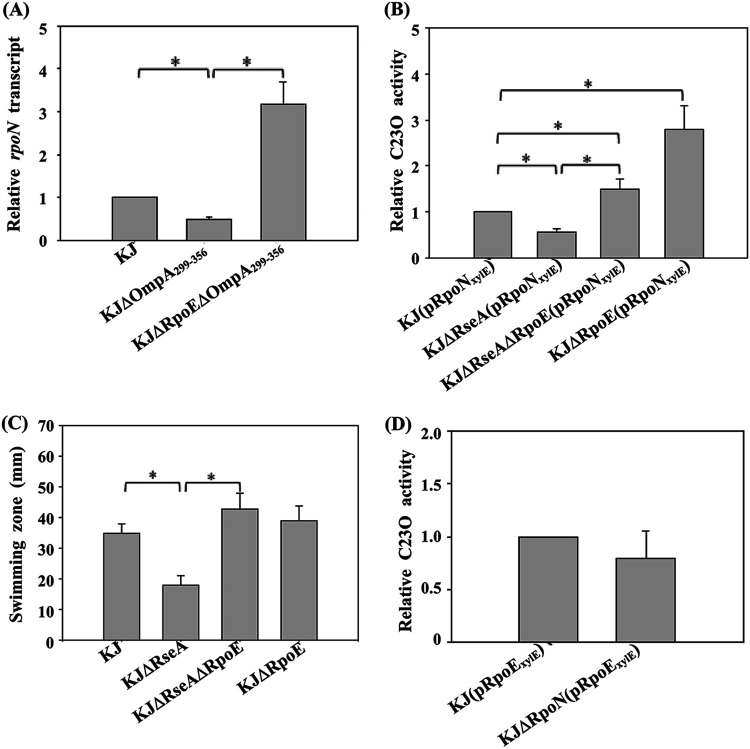
Interplay between σ^E^ and σ^N^. The data represent the means from three independent experiments. Error bars represent the standard deviations for triplicate samples. *, *P < *0.01, calculated via Student’s *t* test. (A) Impact of σ^E^ activation on *rpoN* expression in KJΔOmpA_299–356_. Overnight-cultured bacterial cells were inoculated into fresh LB with an initial OD_450_ of 0.15. After 5 h of aerobic culture, *rpoN* transcript levels were determined by qRT-PCR. All values were normalized to the transcript levels of KJ cells. (B) Impact of σ^E^ activation on *rpoN* expression. Overnight cultures of tested S. maltophilia strains were inoculated into fresh LB medium at an initial OD_450_ of 0.15. After 7 h of culture, their C23O activities were determined. (C) Impact of σ^E^ activation on swimming motility. Logarithmic-phase bacterial suspensions were inoculated into swimming agar (1% tryptone, 0.5% NaCl, and 0.15% agar). The swimming zones were recorded after 48 h of incubation at 37°C. (D) Role of σ^N^ in *rpoE* expression. Overnight cultures of tested S. maltophilia strains were inoculated into fresh LB medium at an initial OD_450_ of 0.15. After 7 h of culture, their C23O activities were determined.

The involvement of σ^E^ and σ^N^ in the Δ*ompA*_299–356_-mediated decrease in MD tolerance was previously established. Next, we explored whether there is an interplay between σ^E^ and σ^N^. First, the *rpoN* transcripts in KJ, KJΔOmpA_299–356_, and KJΔRpoEΔOmpA_299–356_ were determined by qRT-PCR to determine whether activated σ^E^ downregulates *rpoN* expression in KJΔOmpA_299–356_. Compared to wild-type KJ, KJΔOmpA_299–356_ had decreased *rpoN* transcription levels (Fig. 5A). We also observed that the *rpoN* transcript level in KJΔRpoEΔOmpA_299–356_ had reverted to a level higher than the level in wild-type KJ (Fig. 5A). Next, we used a *P_rpoN_-xylE* transcriptional fusion construct (pRpoN_xylE_) to investigate the role of σ^E^ activation in *rpoN* expression in wild-type KJ. Compared to wild-type KJ, KJΔRpoE(pRpoN_xylE_) displayed increased C23O activity (Fig. 5B), in turn indicating that, under a σ^E^-nonactivated condition, free-form σ^E^ exists and exerts a negative impact on *rpoN* expression. We also observed that C23O activity was lower in KJΔRseA(pRpoN_xylE_) than wild-type KJ. Furthermore, the C23O activity of KJΔRseAΔRpoE(pRpoN_xylE_) had reverted to a level even higher than that of wild-type KJ (Fig. 5B), indicating that σ^E^ activation attenuates *rpoN* expression. Collectively, the *rpoN* transcript level was inversely proportional to the free-form σ^E^ level.

To further investigate the correlation between the *rpoN* transcript level and swimming motility, the swimming motilities of KJ, KJΔRpoE, KJΔRseA, and KJΔRseAΔRpoE were evaluated. The swimming motility of KJΔRpoE was slightly, but not significantly, higher than that of wild-type KJ (Fig. 5C). However, KJΔRseA exhibited decreased swimming motility, and *rpoE* deletion from the chromosome of KJΔRseA reverted the swimming motility to the wild-type level (Fig. 5C). Based on these results, we concluded that σ^E^ activation-mediated *rpoN* downregulation results in compromised swimming motility.

Next, we investigated whether *rpoN* regulates *rpoE* expression. The plasmid pRpoE_xylE_, a *P_rpoE_-xylE* transcriptional fusion construct, was transfected into KJ and KJΔRpoN to evaluate C23O activity. KJΔRpoN(pRpoE_xylE_) and KJ(pRpoE_xylE_) exhibited comparable C23O activities (Fig. 5D).

## DISCUSSION

ECF σ factors are crucial in bacterial signaling networks, as they allow bacteria to recognize external signals. ECF σ factors remain in an inactive state under normal conditions, via either nonexpression or functional restriction by the anti-σ factor. In response to stress signals, ECF σ factors are activated by upregulating their expression or sequestering them from the anti-σ factor. Free ECF σ factors recruit the associated RNAP core enzyme, drive the RNAP holoenzyme to bind to specific promoters, and induce gene expression. In the best-known ECF-mediated regulatory circuits, the genes responsible for stress alleviation are generally not expressed under normal conditions, but are upregulated under stressed conditions via ECF σ factor-mediated transcription. In this study, we revealed a particular regulatory circuit mediated by σ^E^. Transcriptome and promoter assays (Table 1; Fig. 4B) revealed that *ompO* is intrinsically expressed. Intrinsic expression of *ompO* should be driven by the housekeeping σ factor, σ^D^. Δ*ompA_299–356_*-mediated stress induced the upregulation of σ^E^, which drove *ompO* expression. σ^E^-mediated *ompO* expression levels may be lower than those mediated by σ^D^. Therefore, *ompO* downregulation in KJΔOmpA_299–356_ may be due to the σ factor switch from σ^D^ to σ^E^.

Based on their structures, OMPs can be mainly classified into two types, namely, the TolC family and β-barrel proteins. The TolC family comprises proteins with an α-helical trans-periplasmic tunnel embedded in the outer membrane via a contiguous β-barrel channel. TolC-like proteins, which are inner membrane-associated periplasmic proteins, usually assemble with integral inner membrane proteins to form a tripartite efflux pump, which is involved in chemical export (41). β-Barrel OMPs, which include OmpA, OmpX, phospholipase A, general porins, substrate-specific porins, and TonB-dependent transporters (42), generally function as channels for the influx or efflux of hydrophilic molecules. Of these β-barrel proteins, OmpA is particularly important, as it tightly attaches the outer membrane to the PG layer via its periplasmic domain (43) and, thus, plays a critical role in envelope stability. Unlike the OmpA protein, most β-barrel OMPs, such as OmpO investigated in this study, are devoid of the periplasmic domain; therefore, most β-barrel OMPs participate in molecule transportation.

A correlation between OMP deletion and a decrease in oxidative stress tolerance has been reported in some bacteria, but most such instances have involved TolC-like OMPs, such as those in Salmonella enterica, Acinetobacter baumannii, Cronobacter sakazakii, and Pseudomonas syringae (44–47). This is likely due to the deleted TolC-like OMPs being members of the tripartite efflux pumps, which extrude the toxic compounds generated during oxidative stress. Therefore, TolC-like OMP deletion compromises oxidative stress tolerance due to the accumulation of toxic compounds. In this study, we found that KJΔOmpA_299–356_ is more susceptible to oxidative stress than its parental strain, KJ. These results seem to indicate that OmpA is an outlet for oxidative-stress-mediating toxic compounds. However, the exact determinant leading to the decrease in oxidative stress tolerance in KJΔOmpA_299–356_ was the downregulation of the expression of another OMP, OmpO, supporting that the biological significance of OmpA is envelope stabilization, rather than molecular transport. OmpA defects cause ESR and σ^E^ activation. OmpO, an intrinsically highly expressed OMP, is a member of the σ^E^ regulon, and its expression is downregulated upon σ^E^ activation. We further established a novel regulatory circuit of Δ*ompA_299–356_*-σ^E^-*ompO* involved in the Δ*ompA_299–356_*-mediated decrease in oxidative stress tolerance.

The association between ECF and oxidative stress adaptation has been widely reported in several microorganisms, such as Porphyromonas gingivalis (σ^E^), Bacteroides fragilis (EcfO), Bradyrhizobium japonicum (CarQ), and Shewanella oneidensis (σ^E2^) (13, 48–50). ECF acts as a positive regulator that protects bacteria from oxidative stress. Under conditions of oxidative stress, ECF is activated and induces ECF regulon expression to address the oxidative stress. Therefore, the loss of ECF function is generally linked to a decrease in oxidative stress tolerance. Here, we demonstrated that Δ*ompA_299–356_*-mediated upregulation of *rpoE* expression exerted a negative effect on oxidative stress tolerance mediated by the downregulation of *rpoN* and *ompO*. OmpO is a novel OMP that is associated with oxidative stress tolerance. *RpoN* is a σ^54^ family σ factor involved in nitrogen assimilation, flagellar motility, type III and VI secretion systems, biofilm formation, and environmental adaptation (51–54). The role of *rpoN* in oxidative stress adaptation has rarely been reported, except in Labrenzia aggregata and Edwardsiella tarda (54, 55). Here, we found that activated σ^E^ had a negative impact on the expression of *rpoN*. Interplay among σ factors is important and widely studied. For example, σ^E^ regulates *rpoN*, *rpoH*, and *rpoD* expression levels in E. coli (24, 56, 57), σ^T^ controls *rpoU* and *rpoR* expression levels in Caulobacter crescentus (15), and *rpoH_II_* expression in Rhodobacter sphaeroides is σ^E^-dependent (58).

The truncated OmpA protein expressed by KJΔOmpA_299–356_ can be stably embedded in the outer membrane but fails to contact with PG (40), which endows KJΔOmpA_299–356_ with pleiotropic defects, including compromised swimming motility (39), increased β-lactam susceptibility (40), and decreased oxidative stress tolerance. Based on our previous study (40) and the novel findings of this study, we conclude that KJΔOmpA_299–356_ experiences PG and envelope stress, leading to upregulation of the σ factors, *rpoP* and *rpoE*, respectively. σ^P^ upregulates the expression of *nagA*, which decreases L1/L2 expression levels and increases bacterial susceptibility to β-lactams (40). In contrast, σ^E^-mediated ESR downregulates *rpoN* and *ompO* expression. OmpO downregulation results in a decrease in oxidative stress tolerance. Furthermore, downregulation of σ^N^ decreases swimming motility (39) and oxidative stress tolerance. Multidrug resistance is a challenging issue for the treatment of S. maltophilia infections, and OmpA represents the most abundant OMP in S. maltophilia. Blocking the interaction between OmpA and PG may present an alternative strategy for S. maltophilia infection control.

## MATERIALS AND METHODS

### Bacterial strains, plasmids, and primers.

The bacterial strains, plasmids, and primers used in this study are listed in Table S5.

### Outer membrane protein preparation and SDS-PAGE.

The purification of outer membrane proteins was carried out as described previously (40). The OMPs were separated by discontinuous SDS-PAGE with a 5% stacking gel and a 15% separating gel. Bands were visualized by staining with 0.1% Coomassie brilliant blue R250 (Bio-Rad) and de-staining with 40% methanol/10% glacial acetic acid.

### Construction of deletion mutant KJΔOmpO.

The deletion mutants were obtained using the double homologous recombination method as described previously (59). The upstream and downstream DNA fragments of *ompO* were gotten by PCR using the primer pairs OmpON-F/OmpON-R and OmpOC-F/OmpOC-R (Table S5). Next, the two PCR amplicons were subsequently cloned into pEX18Tc to yield plasmid pΔOmpO (Table S5). Plasmid pΔOmpO was transferred into S. maltophilia KJ by conjugation. The plasmid’s conjugation, the transconjugants’ selection, and the mutant’s confirmation were carried out as described previously (59).

### Construction of KJL2-OmpOΔOmpA_299–356_ and KJL2-RpoNΔOmpA_299–356_.

Given OmpA is critical outer membrane protein for conjugation, KJΔOmpA_299-356_ failed to obtain a complementation plasmid by conjugation (39). To get the *ompO* and *rpoN* complementation strains of KJΔOmpA_299-356_, KJL2-OmpOΔOmpA_299-356_ and KJL2-RpoNΔOmpA_299-356_ were constructed. Plasmid pEXHH1 (Table S5), constructed in our previous study (40), is designed for cloning the exotic gene intended to be expressed under the L2 promoter drive. The intact *ompO* and *rpoN* genes were amplified by PCR using the primers pairs OmpO-F/OmpO-R and RpoN-F/RpoN-R, and the PCR amplicons were cloned into pEXHH1 to obtain pEXHH1-OmpO and pEXHH1-RpoN, respectively. Plasmids pEXHH1-OmpO and pEXHH1-RpoN were transported into wild-type KJ by conjugation. The *ompO* and *rpoN* genes in pEXHH1-OmpO and pEXHH1-RpoN were inserted into a chromosome downstream from the L2 gene via double homologous recombination to yield KJL2-OmpO and KJL2-RpoN, respectively. The chromosomal *ompA* gene was then deleted from KJL2-OmpO and KJL2-RpoN by double homologous recombination, and KJL2-OmpOΔOmpA_299–356_ and KJL2-RpoNΔOmpA_299–356_ were obtained.

### Construction of the *P_ompO_-xylE* transcriptional fusion plasmid, pOmpO_xylE_.

The DNA fragment containing the promoter region of the *ompO* gene was obtained by PCR using primer pair OmpON-F/OmpON-R (Table S5). The 365-bp PCR amplicon was cloned into pRKxylE, a *xylE* reporter plasmid, yielding pOmpO_xylE_.

### Catechol 2,3-dioxygenase activity determination.

Catechol 2,3-dioxygenase (C23O) is encoded by the *xylE* gene. The C23O activity was measured using 100 mM catechol as the substrate, as described previously (60). The hydrolysis rate of catechol was calculated using 44,000 M^−1^cm^−1^ as the extinction coefficient. One unit of C23O activity (U) was defined as the enzyme amount that converts 1 nmol of substrate per minute. The specific activity was expressed as U/optical density at 450 nm (OD_450_).

### Swimming motility.

The mid-logarithmic-phase bacterial cells were inoculated onto the 0.15% semisolid swimming agar (1% tryptone, 0.5% NaCl, and 0.15% agar). After incubation at 37°C for 48 h, the diameters of the swimming zones (mm) were recorded.

### Statistical analysis.

Student’s *t* test was used for comparison of means between the groups. The Bonferroni correction method was applied to adjust the *P* values.

### Data availability.

The RNA-seq data have been deposited in GenBank under BioProject accession number PRJNA876818.
