# Supplementary material for: Implication of the σE Regulon Members OmpO and σN in the ΔompA299–356-Mediated Decrease of Oxidative Stress Tolerance in Stenotrophomonas maltophilia
Source: Microbiol Spectr. 2023 Jun 7;11(4):e01080-23. doi: 10.1128/spectrum.01080-23 (PMC10433810; doi:10.1128/spectrum.01080-23)
Supplement: Supplemental file 1 — Tables S1 to S5. Download spectrum.01080-23-s0001.docx, DOCX file, 6.0 MB [file spectrum.01080-23-s0001.docx]

**Table S1 The known oxidative stress alleviation systems in *S. maltophilia* KJ**

| Code | Protein |
| --- | --- |
| Smlt2828 | Superoxide dismutase, SodA1 |
| Smlt3238 | Superoxide dismutase, SodA2 |
| Smlt1616b | Superoxide dismutase, SodB |
| Smlt0160 | Superoxide dismutase, SodC1 |
| Smlt0161 | Superoxide dismutase, SodC2 |
| Smlt0372 | Catalase, KatA1 |
| Smlt1385 | Catalase, KatA2 |
| Smlt2537 | Catalase, KatMn |
| Smlt3583 | Catalase, KatE |
| Smlt3183 | Glutathione peroxidase, Gpx1 |
| Smlt3228 | Glutathione peroxidase, Gpx2 |
| Smlt4676 | Glutathione peroxidase, Gpx3 |
| Smlt0840 | Alkyl-hydroperoxide reductase, AhpF |
| Smlt0841 | Alkyl-hydroperoxide reductase, AhpC |
| Smlt1829 | SmeVWX pump, SmeU1 |
| Smlt1830 | SmeVWX pump, SmeV |
| Smlt1831 | SmeVWX pump, SmeW |
| Smlt1832 | SmeVWX pump, SmeU2 |
| Smlt1833 | SmeVWX pump, SmeX |
| Smlt2201 | SmeYZ pump, SmeY |
| Smlt2202 | SmeYZ pump, SmeZ |
| Smlt1537 | MacABCsm pump, MacCsm |
| Smlt1538 | MacABCsm pump, MacBsm |
| Smlt1539 | MacABCsm pump, MacAsm |
| Smlt3976 | Formaldehyde detoxification system, FadB |
| Smlt3977 | Formaldehyde detoxification system, FadC |
| Smlt3978 | Formaldehyde detoxification system, FadA |

**Table S2 Transcriptome analysis of the oxidative stress alleviation associated-genes in wild-type KJ and *ompA* mutant, KJΔOmpA_299-356_**

| Locus | Protein | TPM | | Fold change |
| --- | --- | --- | --- | --- |
|  |  | **KJ** | **KJΔOmpA_299-356_** |  |
| 1. Enzymatic alleviation systems | | | | |
| Superoxide dismutases (SOD) | | | | |
| Smlt2828 | SodA1 | 24.05 | 8.80 | -2.73 |
| Smlt3238 | SodA2 | 5242.21 | 3310.89 | -1.58 |
| Smlt1616b | SodB | 78.49 | 78.38 | -1.00 |
| Smlt0160 | SodC1 | 345.50 | 342.14 | -1.01 |
| Smlt0161 | SodC2 | 765.10 | 987.89 | +1.29 |
| Catalases (Kat) | | | | |
| Smlt0372 | KatA1 | 32.02 | 20.00 | -1.60 |
| Smlt1385 | KatA1 | 132.08 | 56.70 | -2.33 |
| Smlt2537 | KatMn | 9.26 | 5.40 | -1.72 |
| Smlt3583 | KatE | 4.83 | 5.87 | +1.21 |
| Glutathione peroxidases (Gpx) | | | | |
| Smlt3183 | Gpx1 | 137.41 | 87.61 | -1.57 |
| Smlt3228 | Gpx2 | 28.05 | 38.33 | +1.37 |
| Smlt4676 | Gpx3 | 163.39 | 172.98 | +1.06 |
| Alkyl-hydroperoxide reductases | | | | |
| Smlt0840 | AhpF | 153.21 | 141.67 | -1.08 |
| Smlt0841 | AhpC | 1644.43 | 1509.27 | -1.09 |
| 1. Non-enzymatic alleviation systems | | | | |
| Efflux pumps | | | | |
| Smlt1829 | SmeU1 | 2.18 | 4.77 | +2.19 |
| Smlt1830 | SmeV | 2.75 | 8.02 | +2.91 |
| Smlt1831 | SmeW | 1.59 | 0.69 | -2.30 |
| Smlt1832 | SmeU2 | 2.28 | 1.66 | -1.37 |
| Smlt1833 | SmeX | 4.73 | 2.59 | -1.83 |
| Smlt2201 | SmeY | 212.97 | 217.27 | +1.02 |
| Smlt2202 | SmeZ | 84.96 | 83.72 | -1.01 |
| Smlt1537 | MacCsm | 9.35 | 9.37 | +1.00 |
| Smlt1538 | MacBsm | 4.30 | 10.02 | +2.33 |
| Smlt1539 | MacAsm | 14.87 | 15.76 | +1.06 |
| Others | | | | |
| Smlt3976 | FadB | 34.43 | 25.09 | -1.37 |
| Smlt3977 | FadC | 42.08 | 30.66 | -1.37 |
| Smlt3978 | FadA | 89.47 | 53.04 | -1.69 |

^a^TPM, Transcripts Per Kilobase Million

^b^Negative fold changes represent genes that were significantly downregulated in KJΔOmpA_299-356_, whereas positive fold changes represent upregulation in KJΔOmpA_299-356_.

**Table S3 LC-MS/MS analysis of the band A protein spot in Fig. 2**

**>Smlt0387**


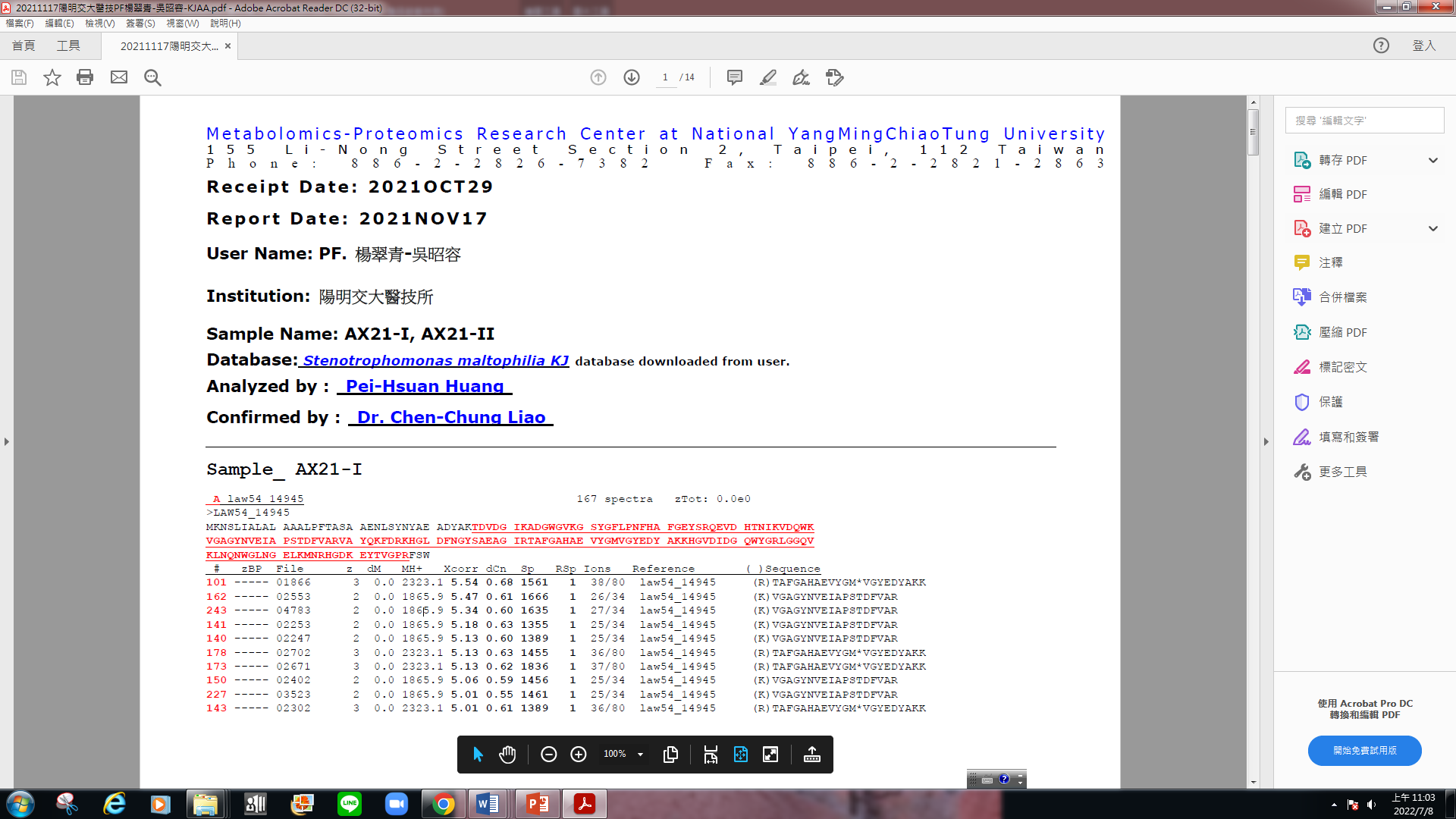

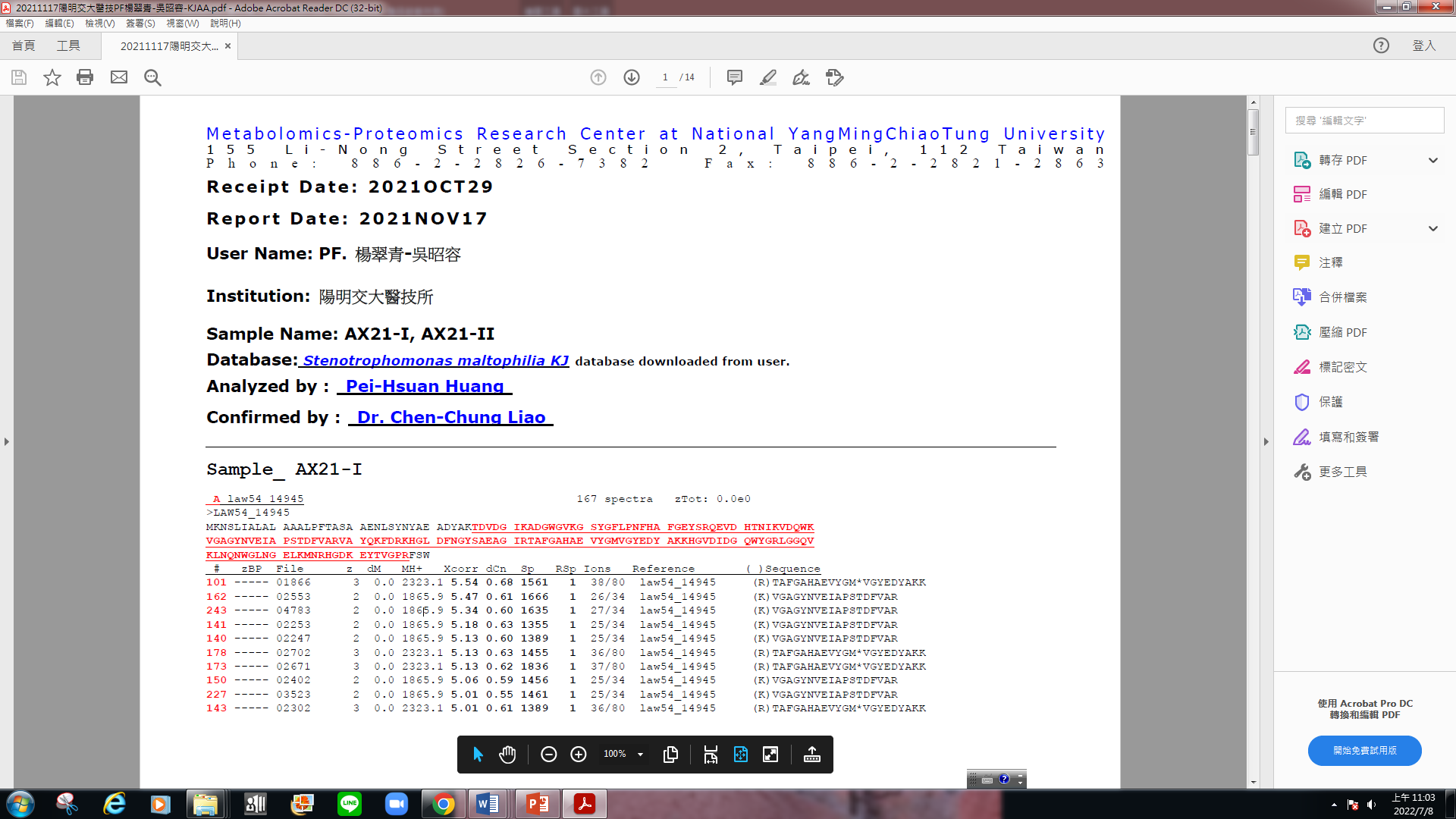

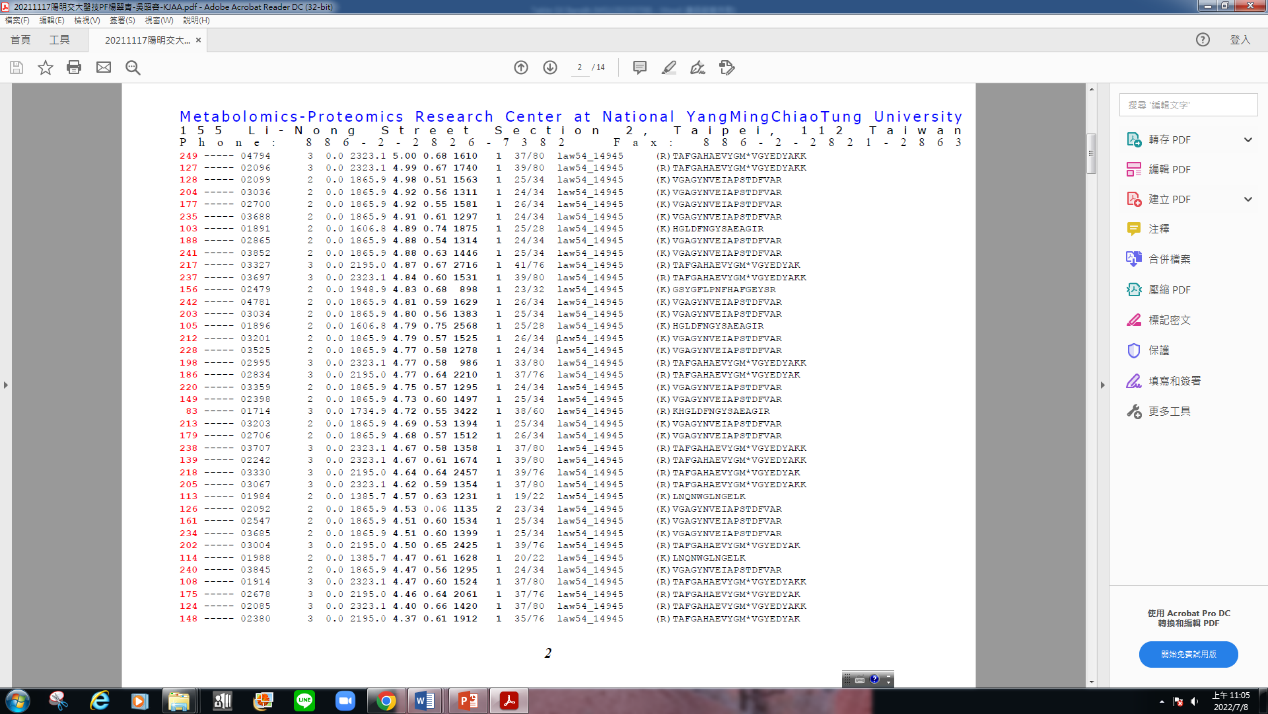

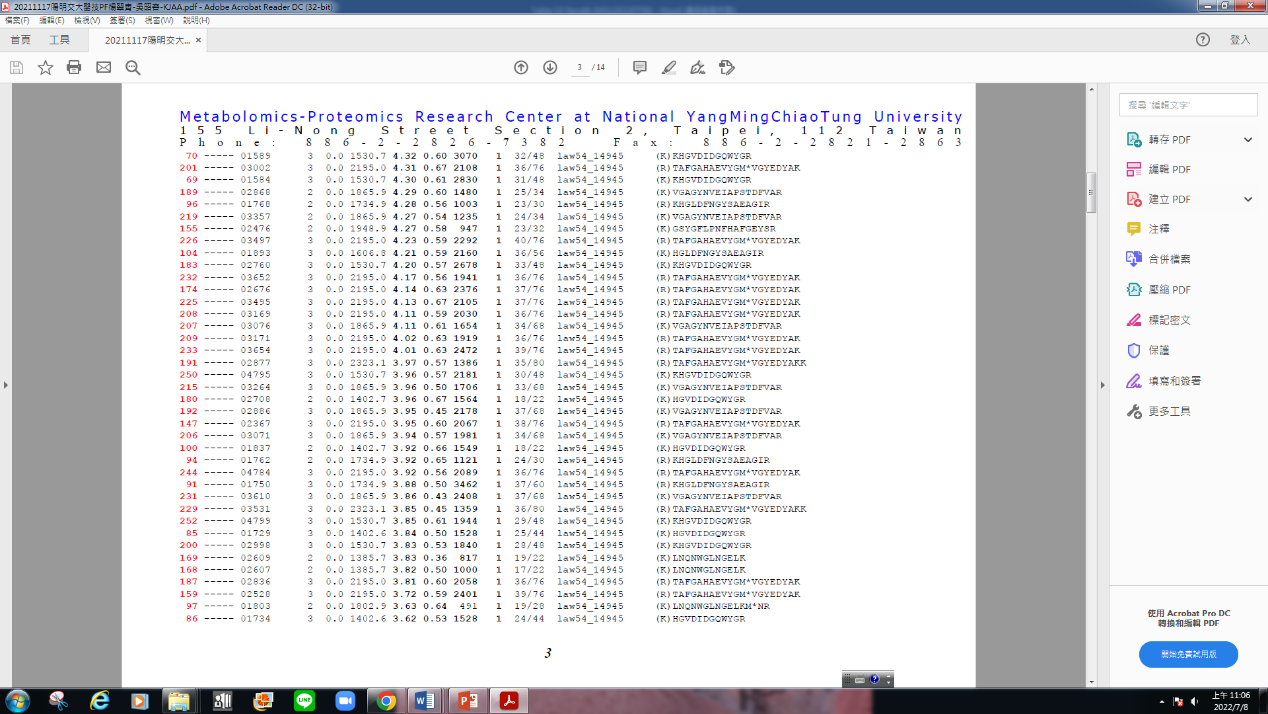

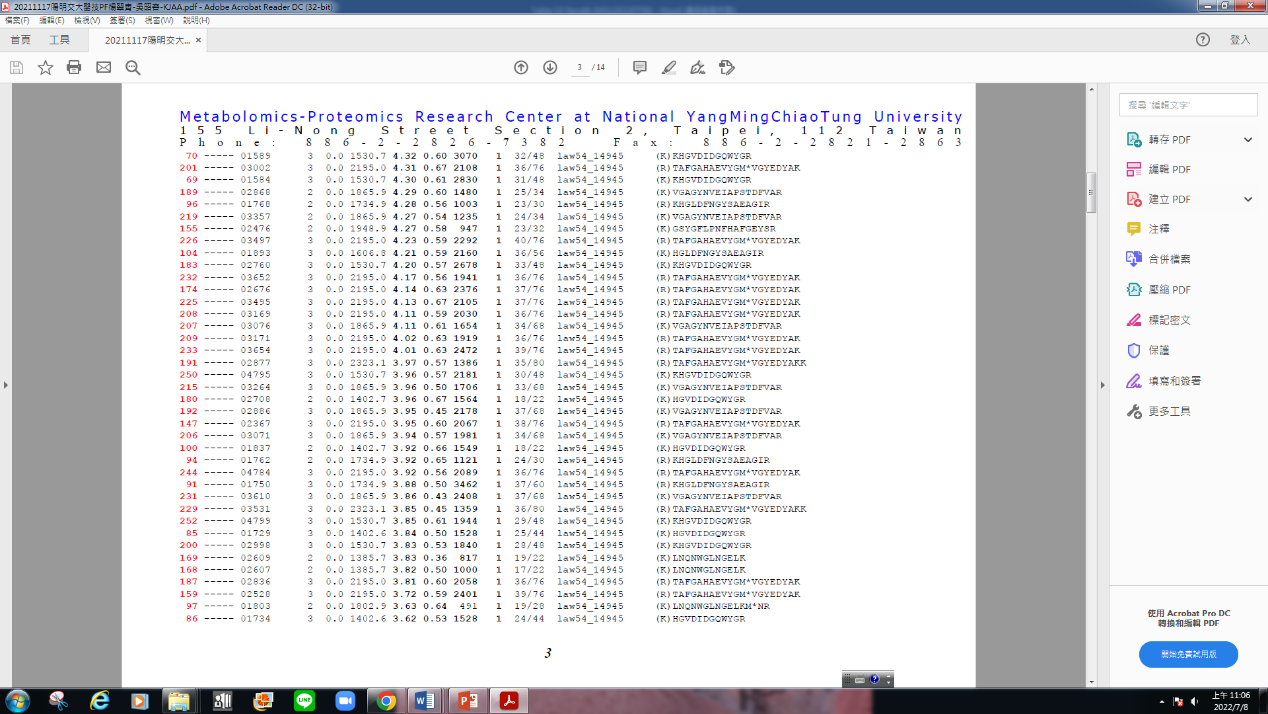

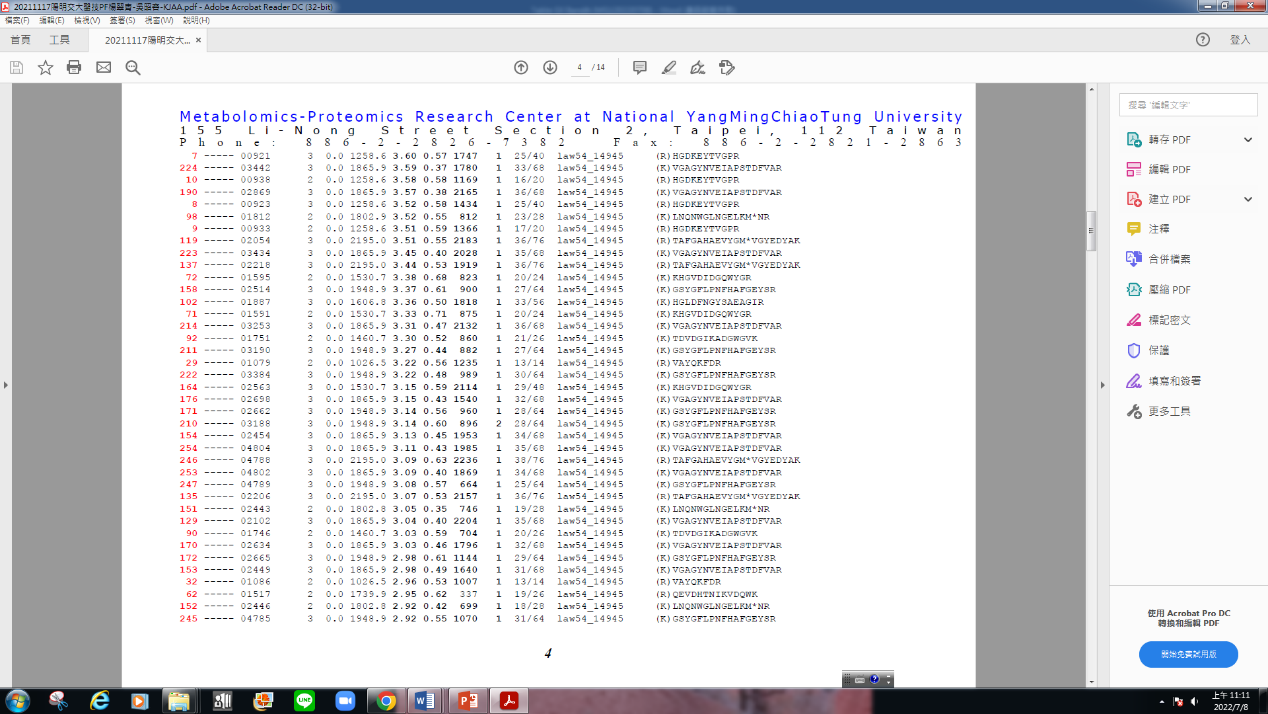

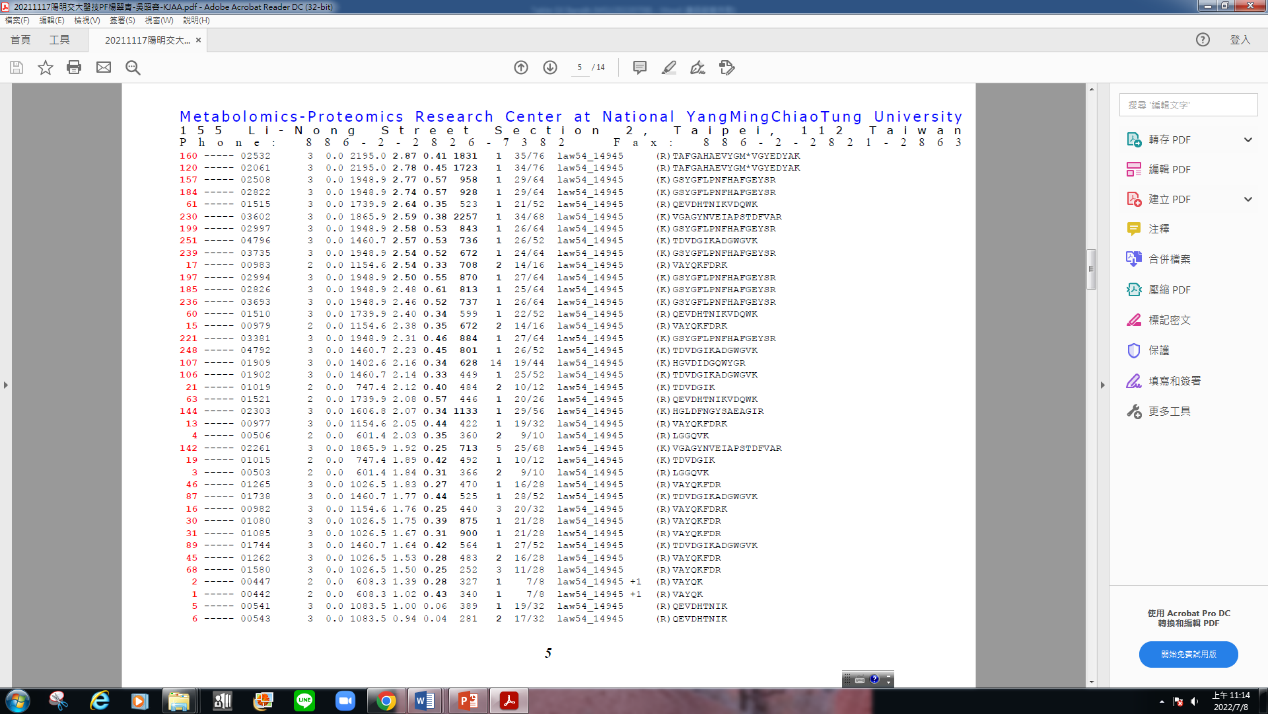


**Table S4 LC-MS/MS analysis of the band B protein spot in Fig. 2**

**
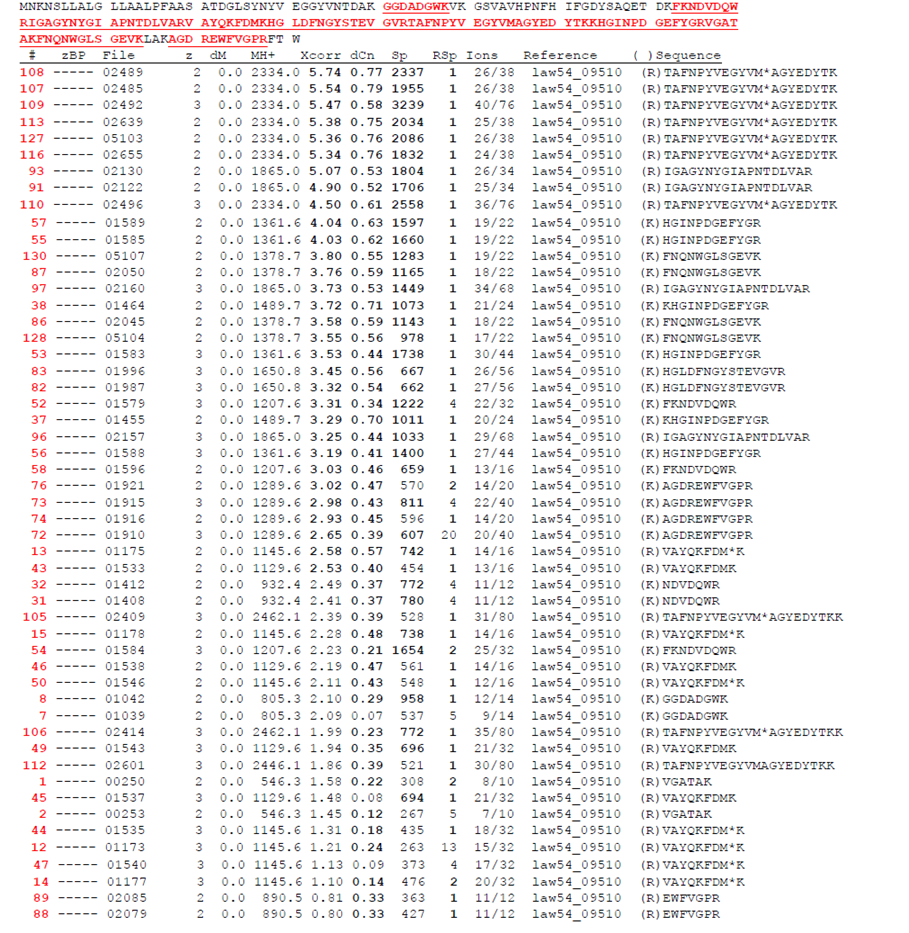
>Smlt0184**

**Table S5 Bacterial strains, plasmids, and primers used in this study**

| Strain, plasmid, or primer | Genotype or properties | Reference |
| --- | --- | --- |
| ***S. maltophilia***  KJ  KJΔOmpA_299-356_    KJΔOmpO  KJL2-OmpAΔOmpA_299-356_  KJL2-OmpAΔOmpA_299-356_  KJΔOmpOΔOmpA_299-356_  KJΔRpoEΔOmpA_299-356_  KJΔRpoPΔOmpA_299-356_  KJL2-RpoNΔOmpA_299-356_  KJΔRseA  KJΔRseAΔRpoE  KJΔRpoN | A clinical *S. maltophilia* isolate  *S. maltophilia* KJ mutant of *ompA* gene; residues 299-356 deleted, named as KJΔOmpA previously  *S. maltophilia* KJ mutant of *ompO* gene  *S. maltophilia* KJΔOmpA_299-356_ with an inserted *ompA* gene downstream L2 gene  *S. maltophilia* KJΔOmpA_299-356_ with an inserted *ompO* gene downstream L2 gene  *S. maltophilia* KJ mutant of *ompA* and *ompO* genes; *ΔompA*, *ΔompO*  *S. maltophilia* KJ mutant of *rpoE* and *ompA* genes; *ΔrpoE*, *ΔompA*  *S. maltophilia* KJ mutant of *rpoP* and *ompA* genes; *ΔrpoP*, *ΔompA*  *S. maltophilia* KJΔOmpA_299-356_ with an inserted *rpoN* gene downstream L2 gene  *S. maltophilia* KJ mutant of *rseA* gene; *ΔrseA*  *S. maltophilia* KJ mutant of *rseA* and *rpoE genes* gene; *ΔrseA, ΔrpoE*  *S. maltophilia* KJ mutant of *rpoN* gene; *ΔrpoN* | 1  2  This study  3  This study  This study  3  3  This study  4  4  2 |
| ***E. coli***  DH5α  S17-1 | F- φ80d/*acZΔM15* Δ(*lacZYA-argF*)*U169* *deoR recA1 endA1 hsdR17* (r_k_^-^ m_k_^+^) *phoA supE44λ* *thi-1 gyrA96 relA1*  λ*pir*^+^ mating strain | Invitrogen  5 |
| **Plasmids** |  |  |
| pEX18Tc | *sacB oriT*, Tc^r^ | 6 |
| pΔOmpO  pΔOmpA_299-356_  pEXHH1 | pEX18Tc with an internal-deleted *ompO* gene; Tc^r^  pEX18Tc with a C-terminus deleted *ompA* gene; named as pΔOmpA previously, Tc^r^  pEX18Tc with C-terminus of *L2* gene and downstream of *L2* gene; Tc^r^ | This study  2  3 |
| pEXHH1-OmpO | pEXHH with an intact *ompO* gene; Tc^r^ | This study |
| pEXHH1-RpoN  pOmpO_xylE_  pRpoN_xylE_  pRpoE_xylE_ | pEXHH with an intact *rpoN* gene; Tc^r^  pRK415 with a *P_ompO_::xylE* transcriptional fusion construct; Tc^r^  pRK415 with a *P_rpoN_::xylE* transcriptional fusion construct; Tc^r^  pRK415 with a *P_rpoE_::xylE* transcriptional fusion construct; Tc^r^ | This study  This study  2  4 |
| **Primers**  OmpON-F  OmpON-R  OmpOC-F  OmpOC-R  OmpO-F  OmpO-R  RpoN-F  RpoN-R | GCGAAGCTTGGCGACAAGAGGGCA  AGGTCTAGAGCAGCCGAAGCGGT  GAATCTAGAGCTGAAGATGAA  GCAGGTACCGGCAGGGAGAAT  GAATCTAGACCAAAAATTCAGCAATG  CCTGAGCTCGGTCAGGTAGCGTTT  GATCTAGAGCAAGATGCGTT  GCGAGCTCGTAAGCTTGTT | This study  This study  This study  This study  This study  This study  This study  This study |

1. Hu RM, Huang KJ, Wu LT, Hsiao YJ, Yang TC. 2008. [Induction of L1 and L2 β-lactamases of *Stenotrophomonas maltophilia*.](https://pubmed.ncbi.nlm.nih.gov/18086856/) Antimicrob Agents Chemother 52:1198-1200.
2. Liao CH, Chang CL, Huang HH, Lin YT, Li LH, Yang TC. 2021. [Interplay between OmpA and RpoN regulates flagellar synthesis in Stenotrophomonas maltophilia.](https://pubmed.ncbi.nlm.nih.gov/34199787/) Microorganisms 9:1216.
3. Li LH, Wu CM, Chang CL, Huang HH, Wu CJ, Yang TC. 2022. [σ^P^-NagA-L1/L2 regulatory circuit involved in ΔompA_299-356_-mediated increase in β-Lactam susceptibility in *Stenotrophomonas maltophilia*.](https://pubmed.ncbi.nlm.nih.gov/36350132/) Microbiol Spectr 10:e0279722.
4. Huang YW, Liou RS, Lin YT, Huang HH, Yang TC. 2014. A linkage between SmeIJK efflux pump, cell envelope integrity, and σ^E^-mediated envelope stress response in *Stenotrophomonas maltophilia*. PLoS One 9:e111784.
5. Simon R, O'Connell M, Labes M, Puhler A. 1986. Plasmid vector for the genetic analysis and manipulation of *Rhizobia* and other Gram-negative bacteria. Methods Enzymol 118:640-659.
6. Hoang TT, Karkhoff-Schweizer RR, Kutchma AJ, Schweizer HP. A broad-host-range Flp-FRT recombination system for site-specific excision of chromosomally-located DNA sequences: application for isolation of unmarked *Pseudomonas aeruginosa* mutants. 1998. Gene 212:77-86.
